# Southern Medical Journals, Etc.

**Published:** 1865-11

**Authors:** 


### SOUTHERN MEDICAL JOURNALS.

It is with no common satisfaction that we receive the promise that at no distant day, our editorial table will again be graced by an exchange list from the Southern States. Thus one after another, and in rapid succession, are the traces of the great conflict passing away, and while all acrimony of feeling seems to be fading from the minds of men North and South, there certainly will be no lack of fraternal spirit between the medical profession of the two portions of our country—for their work has been the same in both armies—simply a labor of kindness to brave and unfortunate men, regardless of the uniform in which they were clad. The question of the practibility of "peaceable secession" has been settled in a way that the blind may see, the deaf hear, and the unlettered read, and the experiment is not likely to be made again. Let us then forget the past, and unitedly address ourselves to the common work before us.

We have received the following notices:—The Richmond *Medical Journal* will be issued at Richmond, Va., commencing in December. It is edited by E S. Gaillard, M. D., W. S. McChesney, M. D. It will be a monthly of 80 or 90 pages. The subscription price being $5, payable in advance.

The Savannah *Journal of Medicine* will be issued from Savannah, Ga., January, 1866. It will be a bi-monthly of 72 pages, at $4 per annum in advance. Editors—Juriah Harriss, M. D., Jas. B. Read, M. D., J. G. Thomas, M. D.

The *Medical and Surgical Monthly*, to be published at Memphis, Tenn. The first number will appear January, 1866. A monthly of 64 pages. Terms for one year, $6; half-year, $4. Editor—Frank A. Ramsey, A. M., M. D.

### RELATIONS OF THERAPEUTICS TO MEDICINE.

It will be admitted by most thinking men that the study of diseased or healthy organization has revealed more of the effects than of the essence of disease. So subtle are the conditions by which the equality of life is preserved, that, in a vast proportion of instances of death, the most refined anatomy and chemistry fail in discovering a commensurate change or in explaining why what was a living creature yesterday, lies before us in a few hours a decomposing mass of clay. Hence, we must be cautious in extensively adopting any therapeutical system which is solely based on inference from visible organic change. In the present imperfect state of our knowledge, we must not neglect that study of therapeutics which is essentially experimental and inductive; and if there be one thing wanting more than another in our science, it is that men should know the nature and difficulties of therapeutic evidence. If, as I have often heard Professor Acland observe, only a few of our well-instructed brethren who are in charge of our public institutions, well aware of the established laws of disease, whether essential or non-essential, and good observers, were to take up any one remedy, whether old or new, say digitalis, and faithfully record on the one hand the character and history of the case, and on the other the results of the use of the particular medicine, or other therapeutical proceeding, we should ere long have such a mass of unbiased statement of facts that safe conclusions could be drawn. Until this is done, the position of therapeutics will be an inferior one. It will not be any trustworthy guide in practice, except in a few salient instances, and will be powerless in its other great function of being the key to, and the test of, pathologic conclusions.

To bring therapeutics up to this level seems to be the great desideratum. We may fairly hold that the time is ripe for the commencement of its study with the view to its higher functions or development. Without placing limits to the material investigations in which we are aided by the microscope and by chemistry, we may believe that our knowledge of the intimate structure and composition of the solids and fluids of the body is so extended as to give to the therapeutist reasons for holding that he is now far better acquainted with the living organism than he was a quarter of a century ago; and that so he has a broader and more secure foundation to build upon. But the therapeutist must also possess assistance of another kind. He must know the principles of accurate reasoning; he must distinguish between the *post hoc* and *propter hoc*; he must be content still to deal with vital phenomena as constituting a class of the nature of which our knowledge is so deficient, that we have still to study their modifications by external agents, experimentally, and without as yet much reference to their relations to structure or to vital chemistry; he must take into account the laws of periodic action in health and in disease, and determine, or seek to determine, as he proceeds, whether the simplest form of acute local as well as of general disease is not under some of these wonderful laws; he must study the question as to whether medicinal interference extinguishes morbid action, postpones it, or, by breaking its circle, as suggested by Professor Boeck, though this be followed by temporary good, deranges the process which is to end in its removal; he must well understand that certainty in medicine must be approached by the balance of probabilities, and have a full insight into the difficulties of medical statistics, which result from the labors of more than one observer. Other circumstances will suggest themselves to you—as the influences of locality, of race, of age, sex, habit, and previous history. I will not dwell on them, further than to remark that, had Broussais attended to one of them, in particular, he would not, I think, have fallen into the error of declaring the non-existence of essential fever from observing disease within a narrow circle of the world.

If therapeutic science is to advance, it must be followed and studied in the most severe scientific spirit.—*Dr. Stokes' Address before Brit. Med. Assoc.*

### ON CHRONIC ULCERS.

By FREDERICK C. SKEY, F. R. S., etc,

The more chronic the ulcer, the larger its size; the more aged the subject, the more remarkable is the influence of opium in effecting its cure. Treat such a case of chronic ulcer of the largest size, having a pale, flat, bloodless base, a high mound of lymph around it, covered by unhealthy integument, the sore pouring out a large quantity of watery ichor, saturating the linen stockings and other appliances—I say, select such a case, occurring in old age; give such a man ten or fifteen drops of tincture of opium night and morning, leave his bowels alone, and observe the base of the sore in five or six days; it will exhibit a number of minute red points, which, daily increasing in number, will rise up in the form and identity of healthy granulations, and cover the entire surface of the ulcer. Contemporaneously with the gradual elevation of the base of the ulcer is the descent of the surrounding eminence, and the commencement of the process of cicatrization. If I desired to select an ulcer on behalf of a student, with a view to illustrate the character of perfect granulations as they appear in a thoroughly healthy example, I would select an ulcer which had been treated by opium in preference to any other. If it be supposed by any man having limited experience in the employment of opium, that any evil to the constitution attaches to the use of that valuable agent, I can only reply that its salutary action on the ulcer is obtained solely through the healthy influence it exercises on the constitution. Judiciously employed, no drug in our pharmacopœia is more innocuous.

---

Deaths by Cholera in New York.—The deaths by cholera in the city of New York, during previous visitations, foot up as follows:

In 1832 there were 3,513 deaths. In 1834 there were 971. In 1849 there were 5,071. In 1852, 374. In 1854, 2,509. In all other years, 137. Total, 12,775.

---

Married—At the residence of the bride's father, George W. Gideon, October 30, by Rev. T. N. McCorkle, Dr. R. T. Richards and Miss Speedy Gideon, and Dr. B. K. Shurtleff and Miss Lydia Gideon, all of this town.

---

Dr. Valentine Mott's estate was valued at $400,000. He bequeathed his anatomical museum to the New York Medical College.

### OHIO STATISTICS OF THE INSANE.

The last report of the Central Ohio Lunatic Asylum gives the occupations of all persons admitted in twenty-six years. In dividing the number of each occupation by the number of the insane it has furnished in that time, we have the following:

| | |
|---|---|
| Speculators | 1 to 24 |
| Artists | 1 " 58 |
| Clergymen | 1 " 84 |
| Students | 1 " 97 |
| Tailors | 1 " 188 |
| Merchants | 1 " 154 |
| Lawyers | 1 " 169 |
| Physicians | 1 " 184 |
| Farmers | 1 " 195 |
| Butchers | 1 " 215 |
| Blacksmiths | 1 " 315 |
| Laborers | 1 " 431 |

The report gives 22 crazy loafers, 23 physicians, 35 clergymen, 1,195 farmers, for the period covered.

We have received the following books from the publishers: Lectures on Inflammation, being the first course delivered before the College of Physicians of Philadelphia, under the bequest of Dr. Mutter. By J. H. Packard, M. D. Philadelphia: J. B. Lippincott & Co. 1865.

Stimulants and Narcotics—Their natural relations with spinal researches on the action of Alcohol, Ether and Chloroform on the Vital Organism. By Francis E. Austie, M. D., M. R. C. P. Philadelphia: Lindsay & Blackiston. 1865.

Researches on the Medical Properties and Application of Nitrous Oxide. By Geo. J. Zeigler, M. D. J. B. Lippincott & Co. 1865.

